# Chuan Huang Fang combining reduced glutathione in treating acute kidney injury (grades 1–2) on chronic kidney disease (stages 2–4): A multicenter randomized controlled clinical trial

**DOI:** 10.3389/fphar.2022.969107

**Published:** 2022-10-03

**Authors:** Ling Chen, Zi Ye, Danjun Wang, Jianlian Liu, Qian Wang, Chen Wang, Bing Xu, Xuezhong Gong

**Affiliations:** ^1^ Department of Nephrology, Shanghai Municipal Hospital of Traditional Chinese Medicine, Shanghai University of Traditional Chinese Medicine, Shanghai, China; ^2^ Department of Nephrology, Shuguang Hospital Affiliated to Shanghai University of Traditional Chinese Medicine, Shanghai, China; ^3^ Department of Nephrology, Minhang Branch of Yueyang Hospital of Integrative Chinese and Western Medicine Affiliated to Shanghai University of Traditional Chinese Medicine, Shanghai, China

**Keywords:** acute kidney injury, chuan huang fang, chronic kidney disease, renal fibrosis, reduced glutathione, traditional Chinese medicine

## Abstract

Lack of effective drugs for acute kidney injury (AKI) grades 1–2 is a crucial challenge in clinic. Our previously single-center clinical studies indicated Chuan Huang Fang (CHF) might have nephroprotection in AKI on chronic kidney disease (CKD) (A on C) patients by preventing oxidant damage and inhibiting inflammation. Reduced glutathione (RG) has recently been shown to increase the clinical effectiveness of high-flux hemodialysis among patients with severe AKI. In this multicenter randomized controlled clinical study, we designed a new protocol to assess the efficacy and safety of CHF combining RG in patients with A on C. We also explored therapeutic mechanisms from renal fibrosis biomarkers. 98 participants were randomly and equally divided into the RG and RG + CHF subgroups. The RG and RG + CHF groups received general treatments with RG and a combination of RG and CHF, respectively. The therapy lasted for 2 weeks. In this study, the primary assessment result was a difference in the slope of serum creatinine (Scr) over the course of 2 weeks. The secondary evaluation outcomes were alterations in blood urea nitrogen (BUN), uric acid (UA), estimated glomerular filtration rate (eGFR), urinary AKI biomarkers, renal fibrosis biomarkers (transforming growth factor-*β*_1_ (TGF-*β*_1_), connective tissue growth factor (CTGF)), and traditional Chinese medicine (TCM) symptoms. Furthermore, vital signs and adverse events (AEs) were observed. Both groups had a slower renal function decline after treatment than before treatment. Compared with RG group, more reductions of Scr, BUN, UA, and better improvement of eGFR were observed in RG + CHF group (*p* < 0.05). Additionally, the levels of urinary AKI biomarkers, renal fibrosis biomarkers, and TCM syndromes were decreased in RG + CHF group versus RG group (*p* < 0.05). No significant between-group differences were observed of AEs. We thus concluded this novel therapy of CHF combining RG might be a useful method for treating A on C patients.

**Clinical Trial Registration:** http://www.chictr.org.cn, ChiCTR2100043311

## 1 Introduction

Acute kidney injury (AKI) is described as a sudden deterioration in renal function that encompasses both structural failure and loss of functionality (Makris and Spanou, 2016). As a serious complication induced by a variety of critical conditions, AKI might result in significant morbidity and death in both the short and long run (Sawhney and Fraser, 2017). Renal replacement therapy (RRT) ought to be initiated as early as feasible in severe AKI (grade 3), however, there is still a lack of effective drugs for AKI grades 1–2 (Gong et al., 2014; Kellum and Ronco, 2016; Meersch et al., 2018).

Chronic kidney disease (CKD) is a combination of chronic illnesses attributed to a variety of indicators, particularly, inflammation, oxidative stress, and metabolic abnormalities (Hoerger et al., 2015; Yan et al., 2021). AKI and CKD are strongly linked to each other. Furthermore, CKD is an unignorable pathogenic factor for the advancement of AKI (He et al., 2017). Studies have shown that the presence of CKD impaired renal function in individuals with AKI and delayed their recovery after AKI (He et al., 2017; Acosta-Ochoa et al., 2019). It is widely recognized that treating AKI on CKD (A on C) is extremely challenging. Renal fibrosis, which is characterized by glomerulosclerosis and tubulointerstitial fibrosis, is a progressive and chronic condition that affects renal function of CKD patients throughout aging (Bhargava et al., 2021; Li et al., 2021). Renal fibrosis is the most prevalent consequence in practically all instances of progressive CKD, which has few therapeutic choices (Djudjaj and Boor, 2019). With regard to treating renal fibrosis, TCM might be a useful alternative treatment option (Zhou et al., 2020).

According to historical records, Chinese physicians utilized traditional Chinese medicine (TCM) to treat AKI during the era when RRT was absent (Li et al., 2019). In China, TCM has been extensively indicated for the management of renal problems (Norgren and Gong, 2018). Chuan Huang Fang (CHF) is a Chinese herbal formulation synthesized by Professor Xuezhong Gong in Shanghai Municipal Hospital of Traditional Chinese Medicine for the treatment of A on C (Tang et al., 2015; Chen and Gong, 2022b). Our previously single-center clinical studies indicated Chuan Huang Fang (CHF) might have nephroprotection in A on C patients via mechanisms of reducing oxidative damage and decreasing inflammatory reactions (Gong et al., 2014; Gong et al., 2020; Gong et al., 2021). Reduced glutathione (RG) has been shown to be beneficial in controlling local inflammation, reducing accumulation of reactive oxygen species, decreasing inflammatory markers, and lowering oxidative stress in the tissues and organs (Matsubara et al., 2019; Zhang and Bai, 2019; Wang and Wu, 2021). Recently, it has been ascertained that RG could increase the therapeutic efficacy of high-flux hemodialysis among patients with severe AKI (Hu, 2015; Yang et al., 2020). Thus, we subsequently optimized the original protocol by utilizing a new drug treatment method involving the combination of CHF and RG.

We undertook a multicenter randomized controlled trial (RCT) for assessing the clinical efficacy and safety of CHF combing RG against A on C. By completing this trial in a timely manner, a unique pharmacological treatment method for AKI grades 1–2 would be developed.

## 2 Materials and methods

### 2.1 Study design

A multicenter randomized controlled clinical trial was performed in this study. The study duration for this research were ranged from 26 December2016- 13 April2020. Approval for the trial was granted by the international review board and ethical committees of each participating hospital and the Ethics Committee of Shanghai Municipal Hospital of Traditional Chinese Medicine (No.2020SHL-KYYS-60). This trial was registered in the Chinese Clinical Trials Register (No. ChiCTR2100043311) before the enrolment of the first participant.

### 2.2 Participants

Three hospitals enrolled participants in this study: 1) Shanghai Municipal Hospital of Traditional Chinese Medicine, 2) Shuguang Hospital Affiliated to Shanghai University of Traditional Chinese Medicine, and 3) Minhang Branch of Yueyang Hospital of Integrative Chinese & Western Medicine Affiliated to Shanghai University of Traditional Chinese Medicine. Overall, 98 participants were classified randomly and equally into the RG and RG + CHF groups. This study enrolled patients who had CKD stages 2–4 complicated with AKI grades 1–2 and toxicity stasis inter-combination syndrome, as well as spleen–kidney qi deficit.

### 2.3 Criteria for inclusion

The following were the criteria for participation in the study: 1) patients satisfied all clinical guidelines for chronic kidney disease stages 2–4 and acute kidney injury grades 1–2, 2) patients satisfied the criteria for a diagnosis for the classification of TCM syndromes, 3) 24 h U-pro of patients ≤2.5 g, 4) ages of patients were range from 18 to 70 years, and 5) patients should be volunteered to participate and signed informed consent.

### 2.4 Exclusion criteria

The following were the criteria for excluding participants from the study: 1) patients who were pregnant or lactating, 2) patients with acute primary illnesses of other organs requiring immediate treatment, including active *tuberculosis*, malignant tumors, or consumption disorders, 3) patients suffering from anorectal disorders who failed to receive enema, 4) patients who had received a kidney transplant, 5) patients who were psychopaths or who had poor compliance, 6) those with an allergic reaction to the therapeutic medication, and 7) those who were enrolled in other clinical trials within the past 3 months.

### 2.5 Criteria for differentiating TCM syndromes

Based on the *Guidelines for Clinical Research of Chinese Medicine (New Drug)* (Zheng, 2002)and *Diagnosis, Syndrome Differentiation and Efficacy Evaluation of Chronic Renal Failure (Trial Protocol)* (He 2006), participants exhibited the following four major symptoms and one to two secondary symptoms, all of which might be indicative of toxicity stasis inter-combination syndrome and spleen–kidney qi deficit. The primary symptoms were: 1) feeling fatigued, having shortness of breath, and being reluctant to talk, 2) a feeling of nausea and vomiting, 3) a dim complexion, and 4) soreness in the knees and waist. The following were the secondary symptoms: 1) discomfort and distention in the abdomen, 2) a loss of appetite, as well as numbness to respond, 3) having skin that is dry and squamous, 4) greasy and thick tongue coating, 5) purple and dark tongue or petechia, and 6) fine tart or slow sunken pulse.

### 2.6 Blinding and randomization

To produce random number sequences, independent biostatisticians used the SPSS (version 21.0) program, which was used to establish these sequences using a simple random approach. The subjects agreed to take part in the research and completed a formal informed consent immediately. Participants also gave their informed permission before being randomly assigned to groups. Participants were assigned a random number and equally classified into two groups by the investigators depending on the order in which they were enrolled in this study: the RG and RG + CHF groups. During the random sampling process, closed envelopes labeled with sequential coding numerals were employed to keep track of allocation information during randomization, and participants, biostatisticians, and investigators were kept blind to the group allocation. Since there was no use of a placebo in the control group, it was not feasible to keep individuals completely unaware of the therapy. It was, however, concealed from all laboratory personnel who were involved in the investigations.

### 2.7 Study interventions

Participants were recruited for the study after providing formal informed permission. They were categorized at random into the RG and the RG + CHF groups. The participants underwent training for study behavior and health to reduce the possibility of loss and ensure that they could follow the experiment without difficulty. Furthermore, they were informed about potential hazards, their rights, and responsibilities, as well as how to deal with an emergency. All of the patients were given a low-protein, high-quality, and low-salt meal plan to follow (protein consumption of 0.6–0.8 g/kg/day). They received basic therapies with the goal of restoring normal water and electrolyte levels as well as acid-base balance abnormalities, controlling blood pressure, improving anemia, and correcting renal bone illnesses. Certain investigators regularly followed up with participants and assisted them to adhere to treatment procedures and undergo tests as per schedule.

The control group received an intravenous injection (IV) of RG 1.8 g mixed with 0.9 percent normal saline or 5 percent glucose in a volume of 250 ml once every day for a period of 2 weeks. The treatment group additionally received 200 ml CHF twice daily for a total of 2 weeks. Simultaneously, they received an enema of the concentrated solution of CHF once a day for 5 days (i.e., 5 times a week), followed by 2 days of rest. RG was obtained from Chongqing Yaoyou Pharmaceutical Co. Ltd. The quality standards of CHF were controlled as follows: 1) All herbal medicines were from Shanghai Municipal Hospital of Traditional Chinese Medicine pharmacy, which were traceable; 2) CHF decoction was produced by the pharmacy; 3) Our team had professional pharmaceutical staff to detect the main monomer content of CHF (emodin and TMP, etc) using fingerprints method regularly. The principal pharmaceutical ingredients of CHF are *Prepared rhubarb (Zhidahuang), Ligusticum wallichii (Chuanxiong), Smilacis glabrae (Tufuling), Coptidis rhizome (Huanglian), Codonopsis pilosula (Dangshen), Salviae miltiorrhizae (Danshen), Rhizoma Pinellinae Praeparata (Zhibanxia), Pericarpium citri reticulatae (Chenpi), Cordyceps sinensis (Chongcaojunsi)*, etc (Gong et al., 2021; Chen and Gong, 2022b). Major composition and action of CHF are demonstrated below (Table 1).

**TABLE 1:** Composition and action of Chuan Huang Fang.

Ingredient	TCM action	Medicinal parts	Dose (g)
Prepared rhubarb (Zhidahuang)	1. Clear heat-fire 2. Promote diuresis 3. Remove jaundice 4. Detoxify	Root	18
Ligusticum wallichii (Chuanxiong)	1. Benefit qi 2. Promote blood circulation	Root	9
Codonopsis pilosula (Dangshen)	1. Invigorate the spleen 2. Replenish qi	Root	9
Salvia miltiorrhiza (Danshen)	1. Promote blood circulation 2. Remove blood stasis 3. Promote tissue regeneration 4. Regulate menstruation	Root	18
Coptidis rhizome (Huanglian)	1. Clear heat 2. Dry dampness 3. Detoxify	Root	3
Smilacis glabrae (Tufuling)	1. Detoxify 2. Calm endogenous wind 3. Promote diuresis 4. Remove turbid poison	Root	18
Rhizoma Pinellinae Praeparata (Zhibanxia)	1. Eliminate dampness and phlegm 2. Lower adverse qi 3. Prevent vomiting 3. Dissolve lumps and resolve masses	Stem	6
Pericarpium citri reticulatae (Chenpi)	1. Regulate qi 2. Dry dampness to tonify the spleen 3. Lower adverse qi 4. Prevent vomiting	Pericarp	3
Cordyceps sinensis (Chongcaojunsi)	1. Tonify deficiency	Sclerotium	3

TCM, traditional Chinese medicine.

Enema were performed by a professional clinician. A clinician took 100 ml concentrated medicine solution of CHF, waiting for the medicine solution close to almost human temperature, and put it into an enema bag. The patient was told to lie on the side of the operating bed, the clinician inserted enema tube to the patient’s anus 20–30 cm deep and then injected the medicine solution slowly, each dripping time for 20 min. After enema operation, the patient was instructed to lie on his pillow with buttocks rising 10–15 cm. The medical liquid should be maintained in the patient’s intestine for more than 1 h.

A series of related studies were carried out, and data were recorded at baseline and every week throughout the duration of therapy. Patient data was gathered, efficacy-related exams were carried out, and indicators of mechanism and safety, as well as medication administration, were meticulously observed throughout the study.

### 2.8 Outcome measures

The primary assessment result was measured and represented as the alteration in the slope of serum creatinine (Scr). The secondary assessment outcomes were post-treatment alterations in blood urea nitrogen (BNU), uric acid (UA), estimated glomerular filtration rate (eGFR), urinary AKI biomarkers including neutrophil gelatinase associated lipocalin (NGAL) and interleukin-18 (IL-18), as well as TCM syndromes. Besides, levels of renal fibrosis biomarkers from serum samples, notably, transforming growth factor-*β*_1_ (TGF-*β*_1_), and connective tissue growth factor (CTGF) were assessed both before and after treatment. Moreover, 1 week before and 2 weeks following the treatment, safety results and vital signs indices (hemoglobin, serum potassium, urine routine, blood routine, and electrocardiogram) were documented for each participant. In the meantime, adverse events (AEs) were continuously observed, precisely documented, and appropriately addressed by researchers throughout the trial.

### 2.9 Evaluation criteria for TCM syndromes

Statistical analysis of the improvement in clinical symptoms score was performed in strict compliance with the Guidelines for Clinical Research of Chinese Medicine (New Drug) (Zheng, 2002). The primary and secondary symptoms of toxicity stasis inter-combination syndrome and spleen–kidney qi deficiency were categorized as mild, moderate, and severe with 2, 4, 8 points, and 4, 8, 12 points, respectively. The two values of total scores generated by the measuring scale depending on TCM symptoms for each patient were added together and employed to compute the efficacy indicator (EI).

EI = (Total symptom score before treatment − Total symptom score after treatment)/Total symptom score before treatment × 100%

The treatment efficacy was evaluated using EI. Symptom improvement levels were defined as follows: clinical control (EI ≥ 95%), significant effect (94% > EI ≥ 70%), and effectiveness (69% > EI ≥ 30%) to inefficacy (EI < 30%).

### 2.10 Determination of the sample size

In this study, we used baseline Scr change to estimate sample size. Premised on previous studies (Zhang and Bai, 2019; Gong et al., 2020), we computed the mean Scr value (160.39 μmol/L), and the standard deviation (44.43 μmol/L). Thus, we hypothesized that the Scr level in the RG + CHF group would be obviously lowered (by over 30 μmol/L) as opposed to that in the control group. SPSS (version 21.0) software was utilized to generate a random number table, and the included participants were randomly and equally divided into the control and treatment groups. It was computed with the help of the sample size estimation program PASS (version 15.0.3) to obtain significant difference *α* = 0.05 (one-sided test) and test efficacy 1–*β* = 0.90. Premised on a 20% drop-out rate, the predicted sample size for random sampling was 49 cases within every group for 98 cases in the study.

### 2.11 Statistical analysis

Regarding the Full Analysis Set (FAS), which is made up of individuals who were randomly assigned into two groups, the principal analysis was carried out. Participants who successfully completed the research protocol and had good compliance were defined as the per-protocol sample. In conformity with the Intend-to-Treat (ITT) principle, all subjects receiving test medications were included in a safety analysis.

Continuous variables were presented as mean ± standard deviation or by the median. In this case, categorical variables were displayed in the form of numbers and percentages. Student’s *t*-tests or one-way ANOVA were applied for continuous data exhibiting normal distribution; in contrast, Pearson’s chi-square test (or Fisher’s exact test for cell count <5 in any cell) was employed for comparisons involving categorical variables. All statistical analyses were conducted using a two-sided design. *p* < 0.05 was established as a determinant of statistical significance. The analyses of statistical data were carried out by a biostatistician who was not engaged in the research with the aid of the SPSS (version 21.0) software package. There were no further analyses or interim analyses carried out in this study.

## 3 Results

### 3.1 Participants’ characteristics

**FIGURE 1:**
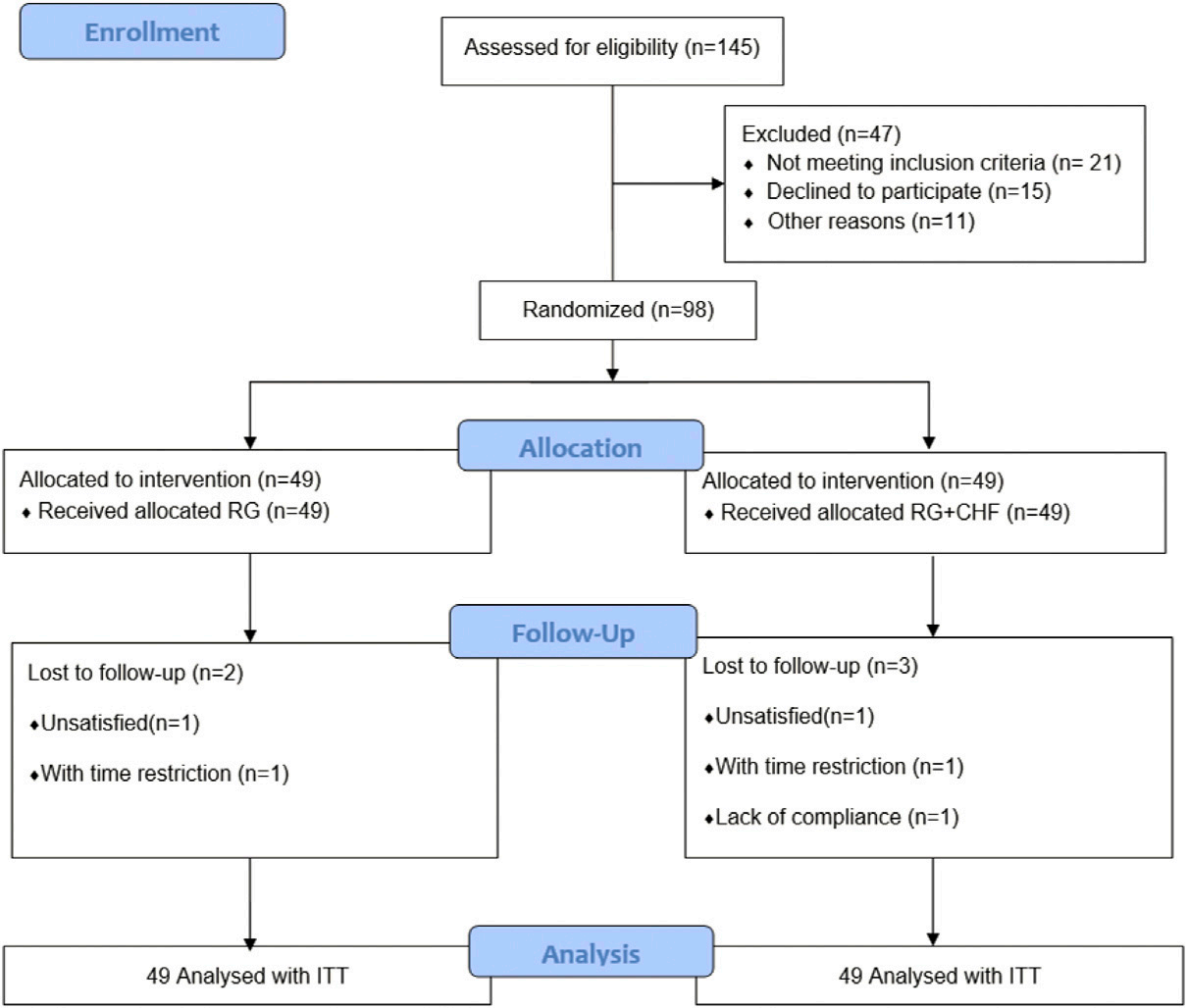
Participants flowchart.

After screening 145 patients, 98 participants were enrolled in the study with 21 patients not meeting inclusion criteria, 15 patients declining to participant and 11 patients for other reasons (Figure 1). RG group included 28 male patients and 21 female participants with an average age of (62.9 ± 9.4) years. In RG + CHF group, there were 30 male participants and 19 female participants with an average age of (61.4 ± 10.5) years. The concomitant diseases were also demonstrated below. According to the statistical results, there were no significant difference in gender, age, and concomitant diseases between two groups (*p* > 0.05). It indicated that the baseline characteristics of two groups were balanced and the efficacy of this clinical study was comparable (Table 2).

**TABLE 2:** Baseline characteristics of the study participants.

Characteristics	Total (*n* = 98)	RG group (*n* = 49)	RG + CHF group (*n* = 49)
Male, *n* (*%*)	58 (59.2)	28 (57.1)	30 (61.2)
Age (years), mean ± SD	62.2 ± 9.9	62.9 ± 9.4	61.4 ± 10.5
Concomitant diseases			
Hypertension, *n* (*%*)	79 (80.6)	41 (83.7)	38 (77.6)
2-Diabetes mellitus, *n* (*%*)	47 (48.0)	23 (46.9)	24 (49.0)

RG, reduced glutathione; CHF, chuan huang fang.

### 3.2 Primary outcome

**FIGURE 2:**
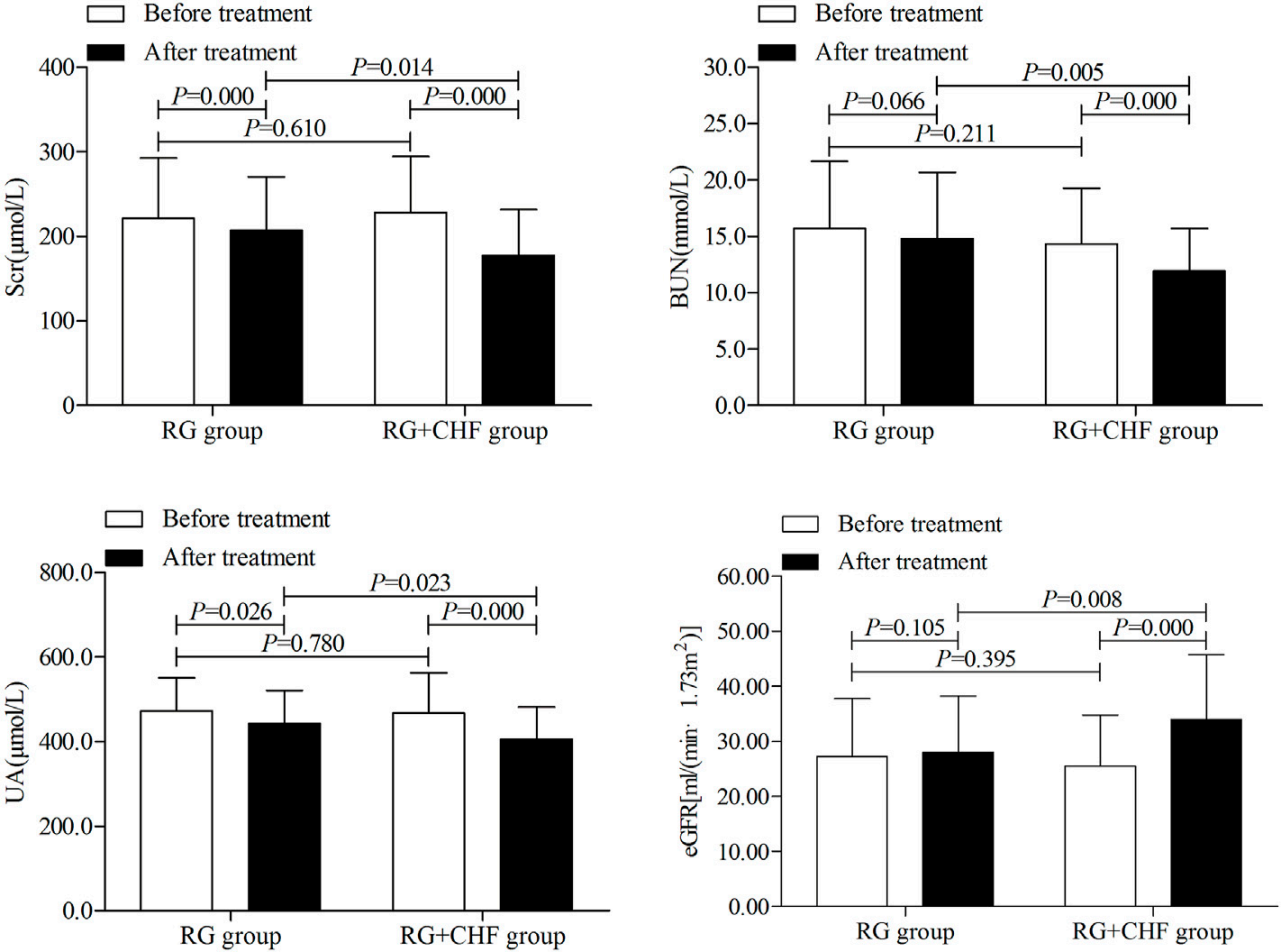
The comparison of renal function indicators between two groups before and after treatment.

Statistical results showed that mean ± SD of the primary outcome Scr after treatment were 207.3 ± 63.4, 177.4 ± 54.6 in RG group and RG + CHF group respectively. Both groups had a significant Scr decline after treatment than before treatment (*p* < 0.01). Compared with RG group, Scr in RG + CHF group was lower and the difference was statistically significant (*p* < 0.05) (Table 3; Figure 2).

**TABLE 3:** The comparison of primary outcome between two groups.

Variable	RG group (*n* = 49)		RG + CHF group (*n* = 49)	
	before treatment	after treatment	before treatment	after treatment
Scr (μmol/L), mean ± SD	221.1 ± 71.7	207.3 ± 63.4*	228.3 ± 66.3	177.4 ± 54.6^*#^

RG, reduced glutathione; CHF, chuan huang fang; Scr, serum creatinine.

∗Statistically significant difference from before treatment; *p* < 0.05 was considered statistically significant.

^#^Statistically significant difference from RG group; *p* < 0.05 was considered statistically significant.

### 3.3 Secondary outcomes

#### 3.3.1 The comparison of renal function indicators between two groups

According to statistical results, it showed that mean ± SD of BUN after treatment were 14.8 ± 5.9,11.9 ± 3.8 in RG group and RG + CHF group respectively. Compared with RG group, BUN in RG + CHF group were lower and the difference was statistically significant (*p* < 0.01). Besides, UA in RG + CHF group were lower and the difference was statistically significant (*p* < 0.05), eGFR was improved and the difference was statistically significant (*p* < 0.01) (Table 4 and Figure 2).

**TABLE 4:** The comparison of secondary outcomes between two groups.

Variables	RG group (*n* = 49)		RG + CHF group (*n* = 49)	
	Before treatment	After treatment	Before treatment	After treatment
Renal function indicators				
BUN (mmol/L), mean ± SD	15.7 ± 5.9	14.8 ± 5.9	14.3 ± 5.0	11.9 ± 3.8*^#^
UA (μmol/L), mean ± SD	472.8 ± 78.4	442.7 ± 79.2*	467.9 ± 94.9	406.6 ± 75.4*^#^
eGFR [mL/(min·1.73m^2^)], mean ± SD	27.3 ± 10.5	28.0 ± 10.2	25.5 ± 9.3	34.0 ± 11.8*#
Urinary AKI biomarkers				
NGAL (ng/ml), mean ± SD	174.0 ± 100.0	138.4 ± 82.3*	177.4 ± 92.0	107.6 ± 42.0*#
IL-18 (pg/ml), mean ± SD	120.5 ± 37.5	104.7 ± 30.8*	126.0 ± 37.4	85.6 ± 27.2*#
Renal fibrosis biomarkers				
TGF-*β*_1_ (μg/L), mean ± SD	187.0 ± 20.8	155.2 ± 21.6*	190.7 ± 25.1	143.9 ± 25.3*#
CTGF (ng/L), mean ± SD	277.1 ± 29.5	232.4 ± 28.1*	281.6 ± 32.4	215.2 ± 37.2*#
TCM syndrome scores	57.9 ± 11.2	34.2 ± 12.0*	57.0 ± 10.4	28.7 ± 10.1^*#^

RG, reduced glutathione; CHF, chuan huang fang; BUN, blood urea nitrogen; UA, uric acid; eGFR, estimated glomerular filtration rate; NGAL, neutrophil gelatinase-associated lipocalin; IL-18, interleukin-18; TGF-*β*_1_, transforming growth factor-*β*_1_; CTGF, connective tissue growth factor.

*Statistically significant difference from before treatment; *p* < 0.05 was considered statistically significant.

#Statistically significant difference from RG group; *p* < 0.05 was considered statistically significant.

#### 3.3.2 The comparison of urinary AKI biomarkers between two groups

**FIGURE 3:**
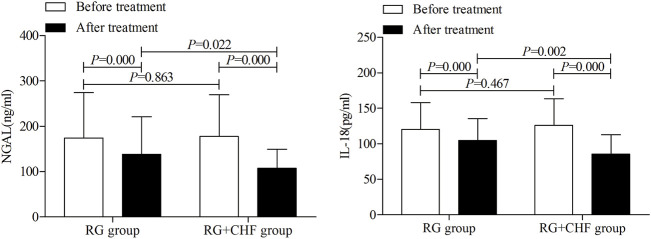
The comparison of urinary AKI biomarkers between two groups before and after treatment.

According to statistical results, it showed that mean ± SD of NGAL after treatment were 138.4 ± 82.3, 107.6 ± 42.0 in RG group and RG + CHF group respectively. Compared with RG group, NGAL in RG + CHF group was lower and the difference was statistically significant (*p* < 0.05). Besides, IL-18 in RG + CHF group was lower and the difference was statistically significant (*p* < 0.01) (Table 4 and Figure 3).

#### 3.3.3 The comparison of renal fibrosis biomarkers between two groups

**FIGURE 4:**
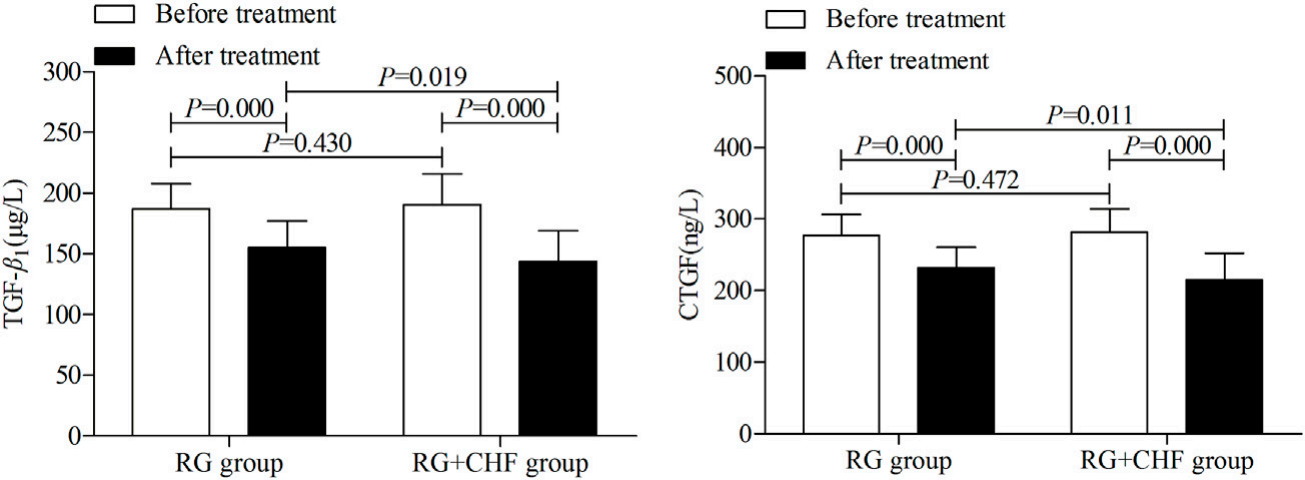
The comparison of renal fibrosis biomarkers between two groups before and after treatment.

Statistical results showed that mean ± SD of TGF-*β*_1_ after treatment were 115.2 ± 21.6, 143.9 ± 25.3 in RG group and RG + CHF group respectively. Compared with RG group, TGF-*β*_1_ in RG + CHF group was lower and the difference was statistically significant (*p* < 0.05). Besides, CTGF in RG + CHF group was lower and the difference was statistically significant (*p* < 0.05) (Table 4 and Figure 4).

#### 3.3.4 The comparison of TCM syndrome scores between two groups

**FIGURE 5:**
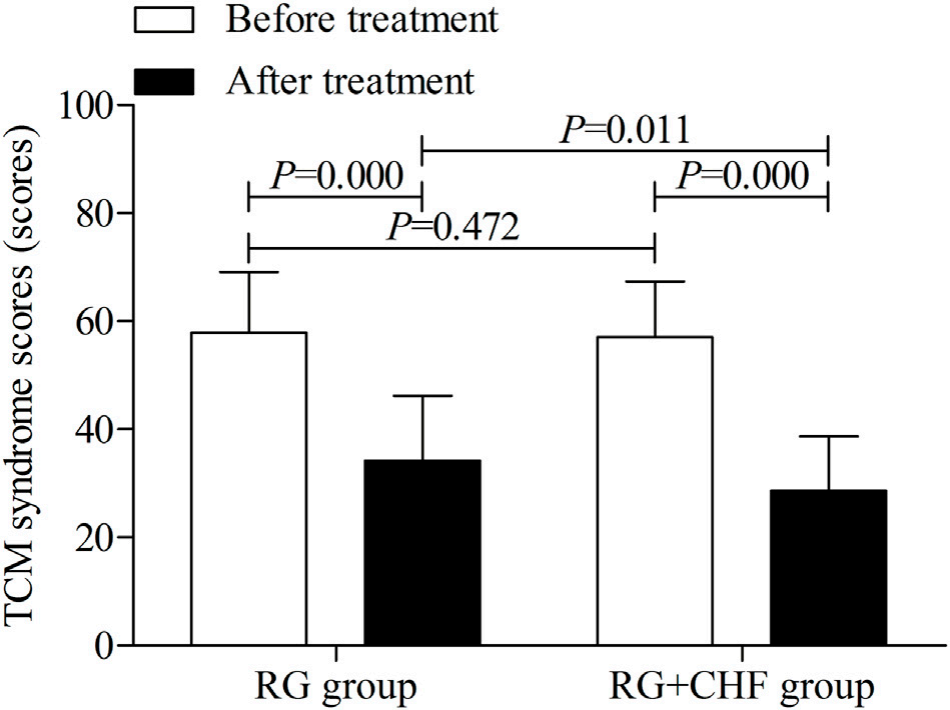
The comparison of TCM syndrome scores between two groups before and after treatment.

Statistical results showed that mean ± SD of TCM syndrome scores respectively were 34.2 ± 12.0, 28.7 ± 10.1 in RG group and RG + CHF group after treatment. Compared with RG group, TCM syndrome scores in RG + CHF group were lower and the difference was statistically significant (*p* < 0.05) (Table 4 and Figure 5).

#### 3.3.5 The comparison of effective rate between two groups

As shown in the table below, significantly effective rate and effective rate in RG group respectively were 12 (24.5%) and 6 (12.2%). While the significantly effective rate and effective rate were 24 (49.0%) and 13 (26.5%) in RG + CHF group. Total effective rate (75.5%) in RG + CHF group were obviously higher than that (36.7%) in RG group, and there was significant statistically difference between two groups (*p* < 0.01) (Table 5).

**TABLE 5:** The comparison of effective rate between two groups.

Effective rate	RG group (*n* = 49)	RG + CHF group (*n* = 49)	Chi-square value	*p* value
Significantly effective, *n* (%)	12(24.5)	24(49.0)	6.323	0.012
Effective, *n* (%)	6(12.2)	13(26.5)	3.199	0.074
Stable, *n* (%)	16(32.7)	10(20.4)	1.885	0.170
Ineffective, *n* (%)	15(30.6)	2(4.1)	12.028	0.001
Total effective rate, *n* (%)	18(36.7)	37(75.5)	14.959	0.000

RG, reduced glutathione; CHF, chuan huang fang.

### 3.5 Safety evaluation

The AEs occurred in the RG and RG + CHF group were demonstrated below (Table 6). There were no SAEs requiring withdrawal reported during the whole treatment period in any group.

**TABLE 6:** Adverse events during treatment period.

Adverse events	RG group (*n* = 49)	RG + CHF group (*n* = 49)
Overall Severe adverse events Gastrointestinal reactions Dizziness or headache Arrhythmia Skin rash Other adverse events	2 0 1 0 0 1 0	3 0 2 1 0 0 0

Adverse events (AEs) were recognized as negative or unpredictable medical manifestations throughout the whole study. Serious adverse events (SAEs) were defined as: 1) critical or life-threatening complications; 2) hospitalization or disability, even death; and 3) other serious hazards and events.

RG, reduced glutathione; CHF, Chuan Huang Fang.

## 4 Discussion

Compared with RG group, more reductions of Scr, BUN, UA, and better improvement of eGFR were observed in RG + CHF group. Additionally, the levels of urinary AKI biomarkers, renal fibrosis biomarkers, and TCM syndromes were decreased in RG + CHF group versus RG group. RG + CHF group demonstrated better renal protective effects than RG subgroup.

AKI is a clinical condition hallmarked by a sudden decrease of renal function, whereas CKD is hallmarked by renal functional or structural impairments. As a consequence of AKI, a significant number of oxygen free radicals are produced, endogenous antioxidants are continually depleted, and high levels of inflammatory substances are secreted, all of which contribute to kidney damage progression (Han and Lee, 2019). Moreover, A on C might be caused by the combination of multiple factors such as decreased prostaglandin synthesis, inflammatory reaction, oxidative stress, abnormal hemodynamics, and increases in the production of thromboxane by the kidney cortex (Gong et al., 2012; Gong et al., 2014; Ruedig and Johnson, 2015; Barnett and Cummings, 2018). Despite the fact that the shift from AKI to CKD has previously been shown in multiple studies, researches of A on C is still in its infancy (Sawhney and Fraser, 2017; Cooper et al., 2018; Bagshaw and Wald, 2021).

According to our published papers (Gong et al., 2014; Gong et al., 2020; Gong et al., 2021), the common precipitating factors of A on C include infection, electrolyte disturbance, hypertension, and stress state, etc. But inflammation and oxidative stress play a crucial role in the underlying molecular mechanisms of such an acute kidney damage. Addressing inflammatory reactions is a viable approach in the treatment of both CKD and AKI (Nikolic-Paterson et al., 2021). AKI can potentially elicit reactions comparable to those observed in CKD, including enhanced cytokine production, higher level of inflammatory cell infiltration, epithelial to mesenchymal transition, and activation of fibroblasts (Lv et al., 2018; Black et al., 2019). Oxidative stress is a condition caused by an imbalance between antioxidants and oxidants (Daenen et al., 2019). Mounting evidence has revealed that oxidative stress performs a fundamental function in the advancement of renal disorders and the progression of kidney-related problems (Zhou et al., 2021). Oxidative stress and inflammation are inextricably related, jointly causing and exacerbating the effects of the other (Luo et al., 2021).

Our previously single-center clinical studies indicated CHF might have nephroprotection in A on C patients (Gong et al., 2014; Gong et al., 2020; Gong et al., 2021). Besides, we published papers about CHF against AKI in animal and cell models as well. Previous research (Gong et al., 2019b) explored clinical dosage of trivalent arsenic inhibition effect and mechanism of renal toxicity. We found CHF could effectively suppressed clinical dose of trivalent arsenic of the kidney toxicity, and its molecular mechanism were associated with the inhibition of caspase three induced renal tubular epithelial cell apoptosis. We also conducted a research on the mechanism of Zhidahuang-Chuanxiong drug pair on tubular epithelial cell apoptosis in contrast-induced acute kidney injury (CI-AKI) rats (Gong et al., 2013). Thus, it was found that activation of p38MAPK pathway played an important role in pathogenesis of CI-AKI, and Zhidahuang-Chuanxiong drug pair might alleviate renal damage in CI-AKI rats through inhibiting the activation of pathway. Based on this result, we further explored the mechanism of the drug pair from nuclear factor erythroid 2-related factor2/Hemeoxygenase-1(Nrf2/HO-1) pathway (Gong et al., 2017). Nrf2/HO-1 pathway was activated and involved in the process, and Zhidahuang-Chuanxiong drug pair could activate and have renal protective effects of CI-AKI rats by inhibiting this pathway. Tetramethylpyrazine (TMP), an active component in both CHF and the medicinal herbs *Ligusticum wallichii (Chuanxiong)*, has the potential to prevent AKI via a variety of processes, including ameliorating oxidative stress damage, suppressing inflammatory responses, deterring apoptotic cell death of intrinsic renal cells, and modulating autophagy (Gong, 2018; Gong et al., 2019a; Gong, 2020; Chen and Gong, 2022a).

Renal fibrosis is by far the most important mechanism that leads to CKD (Gu et al., 2020). It is originally induced by a variety of biophysiological shocks or inflammatory mediators, and it serves as a protective response in the case of kidney injury. Nevertheless, when kidney injuries are prolonged and overreacted, this reaction becomes pathogenic, ultimately contributing to the emergence of ESRD (Inker et al., 2014). TGF-*β*_1_ is the primary profibrotic facilitator in kidney disorders due to its role as a key modulator of fibrosis (Meng et al., 2016). Research findings on the upregulation of active TGF-*β*_1_ have additionally validated the profibrotic function of TGF-*β*_1_ in the etiology of progressive renal fibrosis in a variety of kidney illnesses (Wong et al., 2017; Humphreys, 2018; Kim et al., 2018). Consequently, it has been hypothesized that TGF-*β*_1_ might be a possible treatment target for the clinical management of renal fibrosis. CTGF is an essential component in the onset of renal fibrosis. Although CTGF exists in healthy kidneys as a form of low level, the level is dramatically elevated in a variety of renal illnesses, and it has a substantial impact on the progression of progressive kidney illness (Toda et al., 2018). The gene of CTGF, a matricellular protein, is a straightforward downstream initial response gene of the TGF-*β*_1_ (Lee et al., 2015). According to research results, the treatment group could effectively reduce the level of TGF-*β*_1_ and CTGF, which might provide clinical evidence for early prevention and treatment of A on C patients.

RRT is predominantly applied to treat severe AKI (grade 3). However, there is currently no recognized medication therapy strategy for AKI grades 1–2 (Gong et al., 2014; Kellum and Ronco, 2016; Meersch et al., 2018; Peters et al., 2018). As indicated by the KDIGO recommendations, the primary therapeutic approach for AKI is to regulate the body’s internal milieu, and the vast majority of therapies are supportive rather than curative. Identifying the underlying cause of AKI, maintaining hemodynamic stability, and dealing with severe consequences are all critical throughout the early stages of the disease (Moore et al., 2018). The maintenance of hemodynamic stability needs to be given extensive consideration in AKI because of the impaired autoregulation mechanisms that are present (Ostermann et al., 2019). As previously stated, RG has the potential to attenuate the damaging peroxide metabolites, decrease oxidative stress, and perform specific metabolic functions in the production of inflammatory components (Matsubara et al., 2019; Zhang and Bai, 2019; Wang and Wu, 2021).

Premised on our previously clinical evidence and recent breakthroughs in clinical therapy of A on C, we designed a new protocol with the combination of CHF and RG. And this study tried to explore the medical mechanism of CHF combining RG treating A on C from the aspect of renal fibrosis. The current research would be beneficial in resolving this clinical dilemma in the future. The combination might successfully minimize renal damage of A on C patients, enhance restoration of kidney functionality, and relieve clinical symptoms in these individuals. As a result, we consider that the clinical application values of this therapy method are satisfactory. Based on the findings of this study, we will continue to explore therapeutic effects of CHF combining RG for patients with A on C to gather more relevant clinical data.

## 5 Conclusion

CHF combining RG showed better therapeutic effects than RG alone, and its renal protective effects were associated with reducing Scr, BUN, UA, and improving eGFR, as well as preventing renal fibrosis. We thus concluded that this novel therapy of combining CHF and RG might be a useful method for treating A on C patients.

## Data Availability

The original contributions presented in the study are included in the article/supplementary material, further inquiries can be directed to the corresponding author.
